# In vitro trypanocidal activity of extracts and compounds isolated from *Vitellaria paradoxa*

**DOI:** 10.1186/s12906-023-04175-6

**Published:** 2023-09-28

**Authors:** Guerisson Bairy, Cyrille Oliver Ozzin-Kholy Zolipou, Romaric Nzoumbou-Boko

**Affiliations:** 1 Department of Organic Chemistry, University of Yaoundé 1, B.O Box 812, Yaoundé, Cameroon; 2 Laboratoire de Parasitologie, Institut Pasteur de Bangui, BP 923, Bangui, Central African Republic; 3 Laboratoire des Sciences Biologiques et Agronomiques pour le Développement, Faculté des Sciences, Université de Bangui, BP 1450, Bangui, RCA Central African Republic; 4 Laboratoire de Biochimie, Faculté des Sciences, Université de Bangui, BP 1450, Bangui, RCA Central African Republic

**Keywords:** *Vitellaria paradoxa*, Trypanocidal activity, *In vitro*, *Trypanosoma brucei brucei*

## Abstract

**Background:**

*Vitellaria paradoxa* is used in traditional medicine for the treatment of various diseases in tropical countries; however, nothing is known about its anti-trypanosomal activity. Human African trypanosomiasis is a neglected tropical disease of Sub-Saharan Africa’s poorest rural regions, and the efficacy of its treatment remains a challenge. This study investigates the as-yet-unknown trypanocidal activity of this plant.

**Methods:**

*V. paradoxa*, commonly known as shea tree, was selected for study based on an ethnobotanical investigation. Ultrasonicated extracts from bark and seeds were successively treated with ethyl acetate and water. Column chromatography, NMR spectroscopy and mass spectrometry were used to identify isolated compounds. Purified trypanosomes (*Trypanosoma brucei brucei*) were incubated with serial dilutions of the extracts and isolated compounds at 37 °C in 5% CO_2_ for 24 h. Parasite viability was evaluated under a microscope.

**Results:**

The ethyl acetate extracts of the bark showed the higher in vitro trypanocidal activity against *T. brucei brucei* with median inhibitory concentration (IC_50_) of 3.25 µg/mL. However, the triterpene 1α,2β,3β,19α-tretrahydroxyurs-12-en-28-oic acid and the pentadecanoic acid isolated from the ethyl acetate extract of the seeds showed in vitro trypanocidal activity with IC_50_ of 11.30 and 70.1 µM, respectively.

**Conclusion:**

The results obtained contribute to the validation of the traditional medicinal use of *V. paradoxa*. Our results encourage further investigations of this plant, mainly with respect to its in vivo efficacy and toxicity.

**Supplementary Information:**

The online version contains supplementary material available at 10.1186/s12906-023-04175-6.

## Background

The pharmacological properties of plants used for medicinal purposes for centuries have been confirmed for several diseases [1, 2]. Various plants used in traditional medicine have led to numerous medicine discoveries [3]. These plants represent a rich and largely unknown supply of traditional medication and a bioresource for drugs that merit exploration. The World Health Organization (WHO) has defined medicinal plants as plants containing properties or compounds that can be used in therapy or those that synthesize metabolites to produce drugs and or exert beneficial pharmacological effects on the human or animal body [4, 5].

The WHO affirms that 65 to 80% of the population in developing countries currently use medicinal plants, and mostly in the poorest regions of Sub-Saharan Africa [6, 7]. According to the WHO, about 21,000 plant species have the potential for use as medicinal plants [8, 9]. The people living in rural communities are those who rely the most on traditional medicine for health care [10].

Rural populations are exposed to neglected tropical diseases, including human African trypanosomiasis (HAT), also known as sleeping sickness [11]. This crippling — and potentially fatal disease in the absence of adequate treatment — is still prevalent in Africa, especially in areas of ongoing conflict [12]. However, problems in diagnosis and difficulties in treatments, including safety, administration, toxicity, drug resistance and lost efficacy, drive the need to investigate new anti-trypanosomal agents [13]. The treatment of trypanosomiasis remains a challenge for disease control; therefore, the discovery and development of new therapeutic options are becoming an important priority, especially given that HAT is a neglected disease. In the interest of managing this disease, the use of *V. paradoxa* associated with other plants in HAT treatment may be justified and traditional knowledge must be preserved. During an ethnobotanical investigation in the northern Central African Republic (CAR) on traditional treatments for trypanosomiasis, *Vitellaria paradoxa*, commonly known as shea tree, was as one of the most important medicinal plants used in the preparation of condiments or decoctions or infusions, and was ranked the most important medicinal plant used for this illness.

*Vitellaria paradoxa*, family Sapotaceae, is an important plant species, mainly distributed in West and Central Africa [14]. It is used for its economic interest in the cosmetic industry and for traditional medicine. This plant possesses various features including antioxidant, anti-inflammatory, anti-tumor, and antibacterial properties, but its anti-parasite activity has rarely been investigated [15–17]. Several biomolecules and metabolic derivatives habouring these different properties have been identified and isolated in its extracts [17].

In this study, we highlighted the role of *V. paradoxa* in the traditional medicine practiced in the Batangafo area and tested the in vitro trypanocidal activity of *V. paradoxa* extracts, fractions and isolated compounds.

## Materials and methods

### Materials

#### Ethnobotanical investigation

Ethnobotanical indices (e.g., Relative Frequency of Citation and Informant Consensus Factor) were used for the selection of the plant. *Vitellaria paradoxa* was selected during an ethnobotanical investigation in Batangafo, located in the Ouham province of the CAR, between October 2016 and July 2018. Its anti-trypanosomal activity, mainly in association with other plant extracts, was frequently reported by traditional medicine practitioners and patients living in HAT endemic areas. Plant samples were identified by Pr Olga Yongo, botanist and Director of Center of Study and Research on Traditional African Medicine and Pharmacopoeia (CSRTAMP) at the University of Bangui. The bark and seeds of *V. paradoxa* were collected in this area and transferred to Bangui. A voucher specimen was deposited in the herbarium of CSRTAMP under number No. 054/UB/CER/D.16.

#### Parasite

The Antat 1.1.E. clone of *T. brucei brucei*, originally obtained from the Institute of Tropical Medicine (Antwerp, Belgium) and regularly maintened in the Laboratory of Parasitology of the University of Bordeaux, was used in all the experiments. Frozen parasite vials have been provided by the University of Bordeaux at the Laboratory of Parasitology of the Pasteur Institute of Bangui. After rapid thawings, parasites were cultured three days before use in this study [18, 19]. This experiment did not require the use of animals.

#### Preparation of plant samples

The plant samples (bark and seeds) were air-dried and ground to fine powders for extraction. Extracts were obtained using ultrasonication and by successively adding analytical grade ethyl acetate and water.

## Methods

### Isolation and characterization of compounds

About 1.6 kg of powdered stem bark or seeds was extracted successively by sonication with ethyl acetate and water. Fractionation, or separation, purification, and isolation using the methods described by Eyong et al., 2015, Eyong et al., 2018 and Bairy, 2022 [16, 20, 21].

### In vitro trypanocidal activity assay

The assays were performed according to the procedures previously described [22]. RPMI 1640 growth medium (Eurobio, France) with L-glutamate supplemented with 100 U/mL penicillin, 100 mg/mL streptomycin (Eurobio, France), 25 mM HEPES, 2 mM sodium pyruvate and 10% fetal bovine serum (Dutscher S.A.) was used to test the standard control, plant extracts and compounds. Appropriate dilutions of extracts and compounds (0.5 to 100 µg/mL) were added (100 µL per well) to 96-well tissue culture plates (Falcon, Becton Dickson Labware Europe).

A suspension of 10^5^ purified parasites was then added into each well (100 µL per well) and incubated at 37 °C in 5% CO_2_ for 24 h. The percentage of dead parasites was evaluated by the loss of mobility, considered a characteristic of trypanosome death [23], as observed under a light microscopy at a magnification of X400 (Wilovert Wetzlar A816E, Germany). Pentacarinat (Sanofi Aventis France) was used as the trypanocidal positive control. Activity was expressed in terms of the median inhibitory concentration inhibiting parasite growth by 50% (IC_50_) for each concentration extract and fraction. The mean and standard deviation of IC_50_ were obtained from six repeats.

Some extracts and isolated compounds were poorly solubilized or only solubilized in acetone, a toxic solvent for trypanosomes, and were not used for the in vitro activity test.

## Results

Seven plants were identified from the ethnobotanical survey as being used against HAT. They were *Morinda lucida*, *Khaya senegalensis*, *Vitellaria paradoxa* and *Xylopia aethiopica* with relatively high ethnobotanical indices. *Ricinodendron heudelotii*, *Calancoba welwitchii* and *Terminalia glaucescens* presented the lower ethnobotanical indices. However, in previous studies, in vitro antitrypanosomal activity of the extracts and the isolated compounds of *Morinda lucida* and *Khaya senegalensis* have been demonstrated [24, 25] as well as that of. *Xylopia aethiopica* [26].

In this study, we assessed the in vitro effects of four extracts and four compounds isolated from *V. paradoxa* on *T. brucei brucei* viability. The extracts and isolated compounds had varying degrees of trypanocidal activity. The in vitro screening results for the four extracts indicated that ethyl acetate extract (IC_50_ = 3.25 µg/mL**)** and aqueous extract (IC_50_ = 19 µg/mL) from the bark showed potential trypanocidal activity, while the ethyl acetate extract (IC_50_ = 25.0 µg/mL**)** and aqueous extract (IC_50_ = 36.2 µg/mL**)** of the seeds showed moderate trypanocidal activity., (Table 1).

**Table 1:** In vitro trypanocidal activity of extracts of *V. paradoxa*

Part used	Extract	IC_50_ (µg/ml)
Seed	Aqueous (1)	36.2 ± 3.15
	Ethyl acetate (2)	25 ± 3.1
Bark	Aqueous (3)	19 ± 1.6
	Ethyl acetate (4)	3.25 ± 0.15
Reference compound	Pentamidine	0.086 ± 0.02

Each result is the mean ± SD of IC_50_ for six cultures

**Fig. 1:**
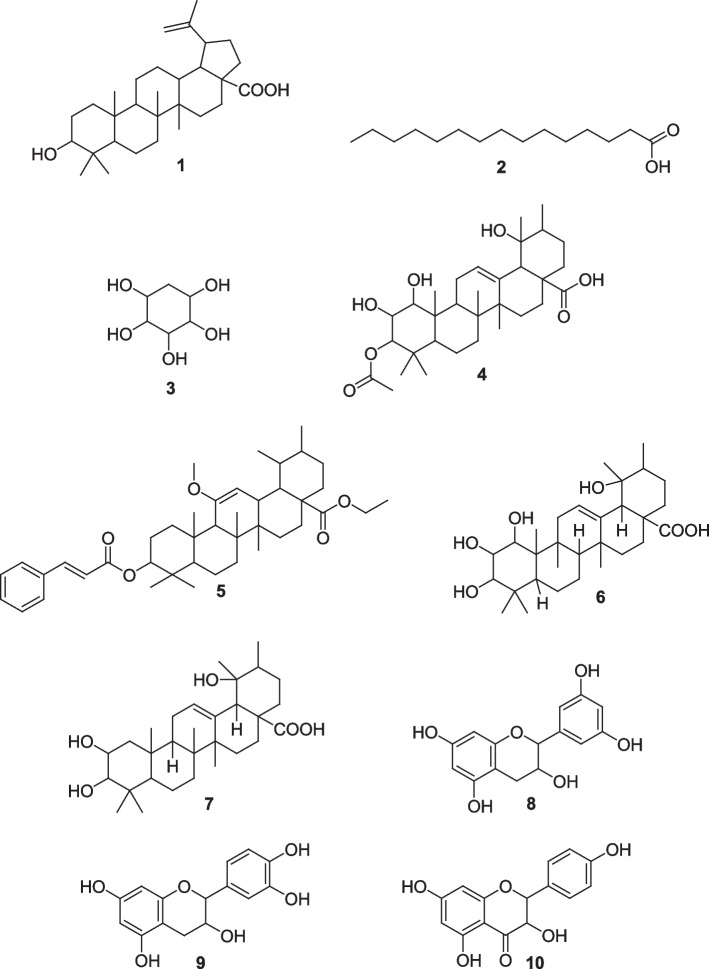
Structures of isolated compounds : Betulinic acid (1), Pentadecanoic acid (2), Cyclitol or cyclohexan-1,2,3,4,5-pentanol (3), 3β-acetoxy-1α,2β,19α-trihydroxyurs-12-en-28-oic acid (4), ethyl3β-(cinnamoyloxy)-11-methoxy-urs-11,12-enoate ester (5), 1α,2β,3β,19α-tretrahydroxyurs-12-en-28-oic acid (6), 2β,3β,19α-trihydroxyurs-12-en-28-oic acid (7), 3,4-dihydro-2-(3’,5’-dihydroxyphenyl)-2-chromene-3,5,7-triol or epicatechin (8), 3,4-dihydro-2-(3’,4’-dihydroxyphenyl)-2-chromene-3,5,7-triol or catechin (9), 2,3-dihydroflavonol or (2R, 3 S)-dihydrokaempferol (10)

Ethyl acetate extracts from bark were fractionated and purified to isolate several compounds, which were characterized as cyclitol (3) and 1α,2β,3β,19α-tretrahydroxyurs-12-en-28-oic acid (6), possessing trypanocidal activity with IC_50_ values of 11.30 and 56.98 µM, respectively (Table 2). The pentadecanoic acid (2) isolate of ethyl acetate seed extracts showed an IC_50_ value of 70.13 µM. Several other compounds were isolated and identified in the ethyl acetate extract, such as betulinic acid (1), 3 β-actoxy-1 α, 2 β, 19 α-trihydroxyurs-12-en-28-oic acid (4) and 2 β, 3β, 19α-trihydroxy-urs-12-en-28-oic acid (7), all belonging to the triterpene class of compounds and other compounds such as ethyl-3β-(cinnamoyloxy)-11-methoxy-urs-11,12-enoate ester (5), 3,4-dihydro-2-(3’,5’-dihydroxyphenyl)-2-chromene-3,5,7-triol (8) or epicatechin (polyphenol), 3,4-dihydro-2-(3’,4’-dihydroxyphenyl)-2-chromene-3,5,7-triol or catechin (9) (flavonoids) (Fig. 1).

**Table 2:** In vitro trypanocidal activity of the compounds isolated of extract

Isolate from	Compound	IC_50_ (µM)
2	Pentadecanoic acid	70.13 ± 1.55
4	1α, 2β,3β,19α-tretrahydroxyurs-12-en-28-oic acid	11.30 ± 2.36
	Cyclotol	56.98 ± 7.12
Reference compound	Pentamidine	0.25 ± 0.058

Each result is the mean ± SD of IC_50_ for six cultures

## Discussion

This study mainly focused on the trypanocidal activity of *V. paradoxa*, selected during an ethnobotanical survey, because it was described as being used in association with other plants to treat trypanosomiasis cases.

A Beninese study on an inventory of traditional recipes used by farmers in the treatment of various pathologies that limit milk production of cows reported that the decoction of *V. paradoxa* bark, associated with *Khaya senegalensis*, *Pseudocedrela kotschyi*, *Parkia biglobosa* and *Afzelia africana* is used to treat animal trypanosomiasis [27]. An *in-vivo* study in Nigeria, has shown that *Tithonia diversolia* and *Vernonia glaberrima* are a potential source of strong anti-*T. brucei* agents [28]. Another study reported the maceration of bark of *V. paradoxa* associated with *Bombax costatum* for treating gastrointestinal diseases and in parasitic diseases in cows [29]. Here, the ethyl acetate extracts of bark, triterpenes and pentadecanoic acid were active against *T. brucei brucei*. Trypanocidal activity was higher in ethyl acetate extracts than in the isolated triterpenes. Ethyl acetate extracts may involve synergy between several compounds.

Previous studies reported that *Vitellaria paradoxa* seeds, leave, bark, and root extracts are composed of several classes of compounds according to extraction solvent [30–33]. A previous study reported that bark of *V. paradoxa* ethyl acetate extract afforded ten pure triterpenoids including the previously undescribed natural products ursaldehyde cinnamate and 11-hydroxy-β-amyrin cinnamate [32]. Additionally, a study showed that shea nut extracts are composed of triterpene alcohol fractions, such as α-amyrin, β-amyrin, lupeol, and butyrospermol [16, 34, 35]. Chromatography screening of the dichloromethane extract of *V. paradoxa* leaves revealed the presence of pentacyclic triterpenic acids among which: Corosolic, maslinic, and tormentic coumaroyl esters and their corresponding triterpenic acids and one isolated triterpenic ester mixture in equilibrium, 3-*O*-*p-E/Z*-coumaroyltormentic acids, showed an attractive promising antitrypanosomal activity [36].

The chemical composition of ethyl acetate extract of *V. paradoxa*, rich in terpenes, is similar atto the chemical composition of *Tithonia diversifolia*, rich in sesquiterpenes, which also proved to have in *vitro* trypanocidal activity [37]. A Kenyan study also showed that sesquiterpene lactones from *Vernonia cinerascens* exert anti-trypanosomal activity in vitro [38]. Similarly, a study on six limonoids, phytochemicals from the triterpenoid class of compounds, reports their anti-trypanosomal activity in vitro against *T. brucei brucei* [39].

Our findings therefore add to the list of the antiparasitic activities of *V. paradoxa* and other plants used against *T. brucei*. In addition, *V. paradoxa* possesses anti-inflammatory and antioxidant proprieties, demonstrated in several studies [15, 16]. These properties may also justify its use in traditional medicine for the treatment of HAT, because excessive pro-inflammatory cytokine production is a hallmark of HAT [40], and increased levels of inflammatory mediators, such as tumor necrosis factor (TNF)-α, are correlated with HAT disease severity [41, 42]. However, current drugs used in trypanosomosis (pentamidine, eflornithine, nifurtimox, and fexinidazole) were alkaloid-like structures. Therefore, the research on the trypanocidal activity of the terpenoids will contribute to the discovery a new therapeutic target and the improvement of treatment.

## Conclusion

Our results contribute to the validation of the traditional use of *V. paradoxa* and tested in vitro plant extracts for trypanocidal activity for the first time. They corroborate its ethnopharmacological use and add to the list of the medicinal properties of this species. These findings encourage further investigations on this plant, mainly for its in vivo efficacy and toxicity. These extracts and fractions may be a novel source for the development of new anti-trypanosomal candidates, alone or in association with other molecules.

### Supplementary Information

**Additional file 1.**

**Additional file 2.**

## Data Availability

The datasets supporting the results of this article are included within the article. However, the raw data used for analysis is contained in supplementary materials.

## Declarations

This was an in vitro study; therefore no animals were used in the study.
