# Supplementary material for: In vitro trypanocidal activity of extracts and compounds isolated from Vitellaria paradoxa
Source: BMC Complement Med Ther. 2023 Sep 28;23:346. doi: 10.1186/s12906-023-04175-6 (PMC10540432; doi:10.1186/s12906-023-04175-6)
Supplement: Supplementary file 1 — Additional file 1. [file 12906_2023_4175_MOESM1_ESM.docx]

**Supporting information.**

1. **Betullinic acid**

**Spectral data of VP-Me**

1H VP5

DEPT

DEPT 90

COSY

HSQC

HSQC TOCSY VP5

HMBC

NOESY VPS

TOCSY VP5

ROESY VP5

VP5-Me
