# Supplementary material for: In vitro trypanocidal activity of extracts and compounds isolated from Vitellaria paradoxa
Source: BMC Complement Med Ther. 2023 Sep 28;23:346. doi: 10.1186/s12906-023-04175-6 (PMC10540432; doi:10.1186/s12906-023-04175-6)
Supplement: Supplementary file 2 — Additional file 2. [file 12906_2023_4175_MOESM2_ESM.docx]

**SUPPORTING INFORMATION**

1. **Composé VP3 : Acide bétulinique**

1. **Composé VP18 : Acide pentadécanoïque**

1. **Composé VP6 : Cyclotol**

1. **Composé VP11 : acide 3β-acétoxy-1α,2β,19α-trihydroxyurs-12-en-28-oïque**

1. **Composé VP2 : ester 3β-(cinnamoyloxy)-11-méthoxy-urs-11,12-enoate d’éthyle**

1. **Composé VP4 : l’acide 1α,2β,3β,19α-tretrahydroxyurs-12-en-28-oïque**

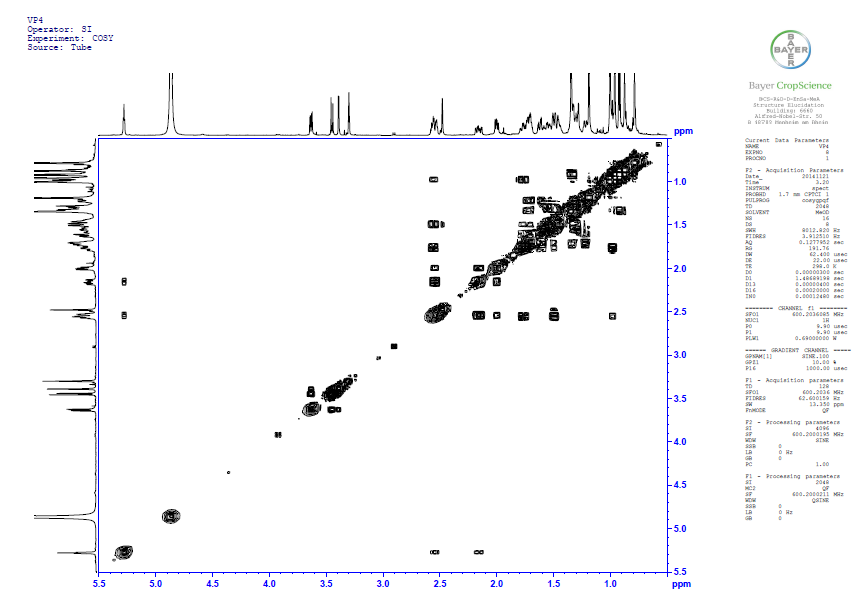

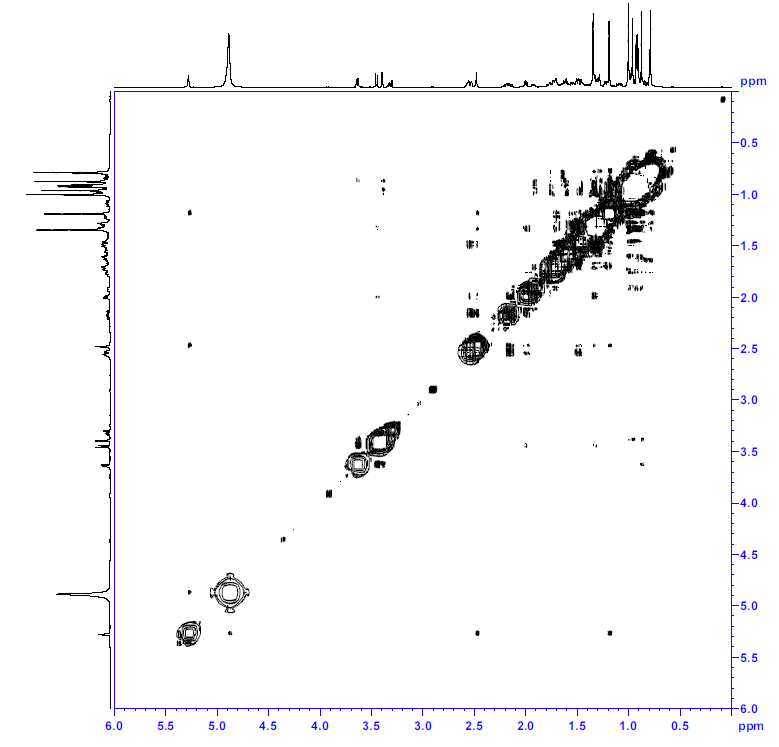

1. **Composé VP5 : l’acide 2β,3β,19α-trihydroxyurs-12-en-28-oïque**

1. **Composé VP5-1 : 3,4-dihydro-2-(3’,5’-dihydroxyphenyl)-2-chromène-3,5,7-triol** ou épicatéchine


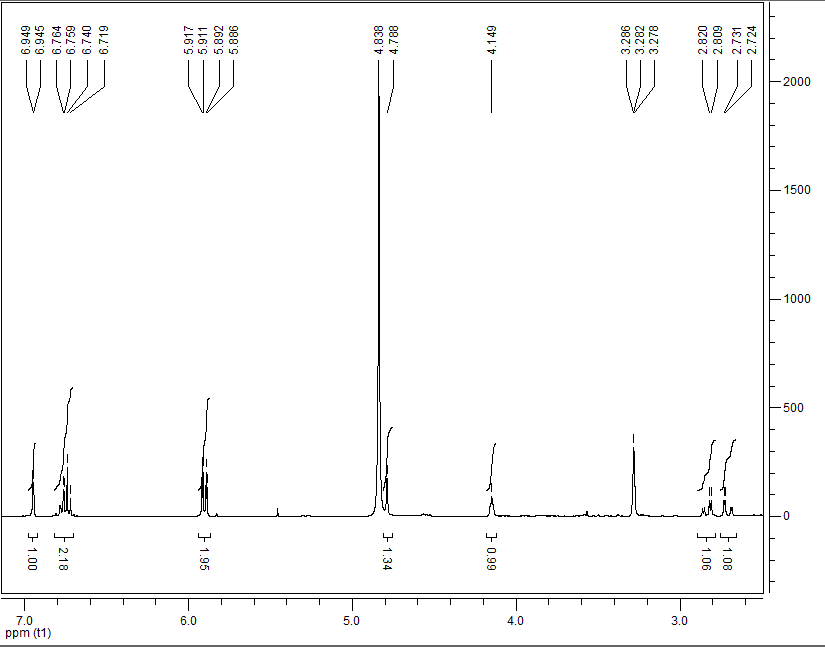


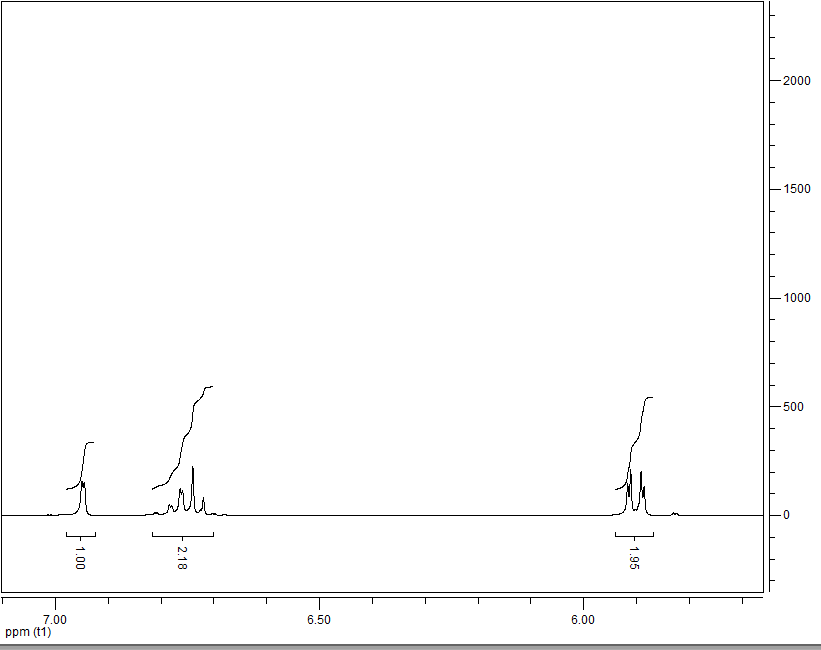


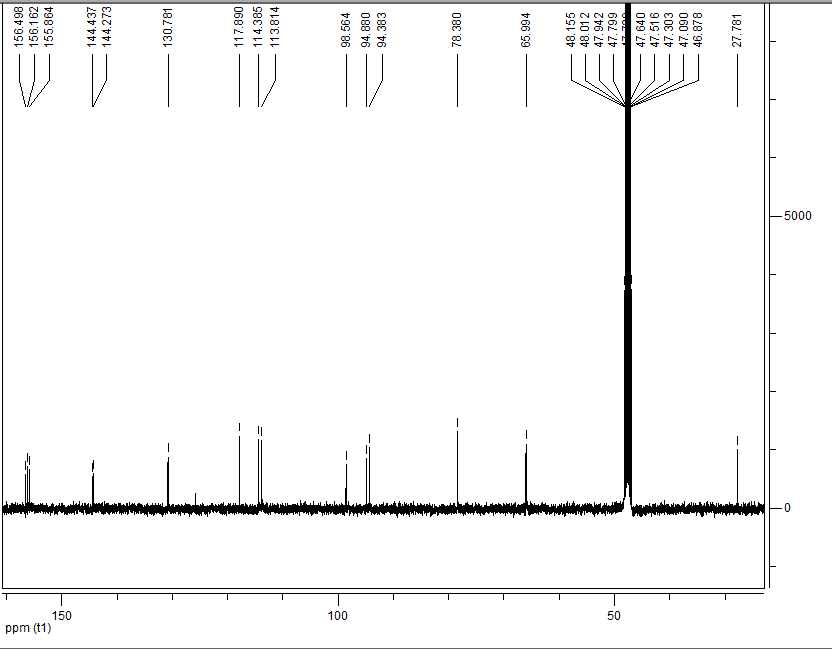

1. **Composé VP7 : 3,4-dihydro-2-(3’,4’-dihydroxyphényl)-2-chromène-3,5,7-triol** ou catéchine

1. **Composé VP17 : 2,3-dihydroflavonol ou (2R, 3S)-dihydrokaempférol**
